# Building research capacity for child and adolescent mental health in Africa

**DOI:** 10.1186/s13034-016-0119-2

**Published:** 2016-09-01

**Authors:** Olayinka Olusola Omigbodun, Myron Lowell Belfer

**Affiliations:** 1 Centre for Child and Adolescent Mental Health, University of Ibadan, Ibadan, Nigeria; 2 Department of Psychiatry, College of Medicine, University of Ibadan & University College Hospital, Ibadan, Nigeria; 3 Boston Children’s Hospital, Harvard Medical School, Boston, MA USA

There is an urgent need to address the wide disparity between scientific research on Child and Adolescent Mental Health (CAMH) emanating from high-resource settings, and low-resource settings like Africa [1–4]. Africa has a largely youthful population with over 50 % children and adolescents, many of whom live in ‘Exceptionally Difficult Circumstances’ with increased risk for mental disorders [5, 6]. The challenge is to develop both research capacity and to then have the relevant research published in widely disseminated journals.

Despite the identified need, very few resources for CAMH are available [1]. Until recently, there were no formal training programmes for CAMH in sub-Saharan Africa with the exception of South Africa [6]. Similarly, the CAMH research base that should help drive advocacy for service development and resources is absent as seen by the virtual absence of CAMH publications in the international literature [3, 4]. Several African countries do not have a single publication in the field of CAMH [4]. In African settings where publications on CAMH research have trickled out, the lead authors and researchers are usually based in offshore high-resource settings, a situation that limits the opportunity for sustained interventions and impact. The dearth of CAMH research impedes CAMH policy development as well as the implementation of evidence-based practices [2, 4] urgently needed in Africa.

At the close of the era of the Millennium Development Goals (MDGs), Africa had the world’s largest absolute decline in child mortality, meaning that social and health interventions now have to focus on creating a mental health-promoting environment for youth to achieve their potential in this changed environment [7]. In response to the dearth of CAMH resources in Africa, in January 2011 the John D. and Catherine T. MacArthur Foundation approved a grant for “Building Child and Adolescent Mental Health (CAMH) capacity in Africa” through the University of Ibadan, Nigeria. This was to be executed primarily through the development and implementation of a Master of Science degree programme in Child and Adolescent Mental Health (MSc. CAMH). A Centre for Child and Adolescent Mental Health (CCAMH) (www.ccamhi.ui.edu.ng) is now established at the University of Ibadan with guest faculty from institutions on the four continents of Asia, Africa, Europe, and North America.

A major point made in the proposal was that through the research activities of course participants in the MSc. CAMH programme, there would be the gathering of country-specific epidemiological data about the prevalence and nature of mental health problems in children and adolescents, causative pathways, ethnic differences, service utilization, effective interventions that are culture-specific, and the cost effectiveness of treatment programs from which public policy and services can be developed [2]. Further, the MSc. Programme would develop a cadre of professionals who would be a resource to sub-Saharan Africa and offset the pernicious effects of “brain drain.”

From January 2013 when the first set of students commenced academic work to June 2016, three sets of students have completed the 18-month programme with each of the 43 students from the African countries of Ghana, Liberia, Kenya, Nigeria and Sierra Leone executing a research project from primary data. Today, CCAMH has 43 research projects implemented by its graduates and there is support for concerted efforts to get each research project published in a reputable open access journal. CAPMH is the official journal of the International Association for Child and Adolescent Psychiatry and Allied Professions (IACAPAP). It has a similar purpose to the IACAPAP textbook of Child and Adolescent Mental Health, which is evidence-based and open access, thereby accessible to resource-poor Africa and providing information to empower and strengthen CAMH [8]. It is important to ensure the results of CCAMH’s research are disseminated widely and able to inform policy and planning for the health and development of infants, children and adolescents in Africa and beyond.

In this African series, of the four articles published at this first stage, three are pilot interventional studies, and all four have African CAMH professionals as the lead authors. One study showed the need to screen mothers of children with psychopathology as the mothers were found to have high rates of depression. Interventions for children with disabilities were used in studies which showed improvements in the social skills of the pupils with severe intellectual disability after an 8-week social skill intervention, as well as, the effect of a parent-mediated behavioural intervention to reduce aggression in children with autism spectrum Disorder (ASD). The aggressive behaviour of primary school children was mitigated following a group-based problem solving intervention.

These pilot studies by African CAMH professionals provide an avenue for advocacy to policy makers to facilitate the development of resources that could benefit a large number of children. This first series shows the potential that could be tapped into and the promise of these research projects to bring CAMH care to children in resource-poor settings. Lastly, the ability of these researchers to see the fruits of their research reach a CAMH community outside Africa brings enhanced self-esteem and an incentive to mature research skills and persist in an Africa-based professional career.

## Abbreviations

CAMH: child and adolescent mental health
MDGs: millennium development goals
MSc. CAMH: Master of Science in Child & Adolescent Mental Health
CCAMH: Centre for Child and Adolescent Mental Health
IACAPAP: International Association for Child & Adolescent Psychiatry & Allied Professions
ASD: autism spectrum disorder
